# Poly[[triaqua­(μ_3_-4-oxidopyridine-2,6-dicarboxyl­ato)thulium(III)] monohydrate]

**DOI:** 10.1107/S1600536811007628

**Published:** 2011-03-09

**Authors:** Zhu-Qing Gao, Dong-Yu Lv, Jin-Zhong Gu, Hong-Jin Li

**Affiliations:** a School of Chemistry and Biology Engineering, Taiyuan University of Science and Technology, Taiyuan 030021, People’s Republic of China; b Key Laboratory of Nonferrous Metal Chemistry and Resources Utilization of Gansu Province, College of Chemistry and Chemical Engineering, Lanzhou University, Lanzhou 730000, People’s Republic of China

## Abstract

In the title coordination polymer, {[Tm(C_7_H_2_NO_5_)(H_2_O)_3_]·H_2_O}_*n*_, the Tm^III^ atom is eight-coordinated by a tridentate 4-oxidopyridine-2,6-dicarboxyl­ate trianion, two monodentate anions and three water mol­ecules, forming a distorted bicapped trigonal–prismatic TmNO_7_ coordination geometry. The anions bridge adjacent Tm^III^ ions into double chains. Adjacent chains are further connected into sheets. O—H⋯O hydrogen bonds involving both coordinated and uncoordinated water mol­ecules generate a three-dimensional supra­molecular framework.

## Related literature

For the structures and properties of lanthanide coordination compounds including the isotypic Dy and Eu analogues, see: Qin *et al.* (2011 ▶); Lv *et al.* (2010 ▶); Gao *et al.* (2006 ▶). For structures of complexes containing eight-coordinate Tm^III^, see: Qin *et al.* (2011 ▶); Tian *et al.* (2009 ▶).
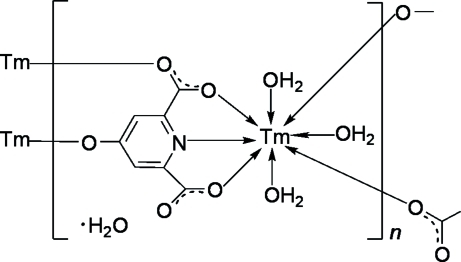


## Experimental

### 

#### Crystal data

[Tm(C_7_H_2_NO_5_)(H_2_O)_3_]·H_2_O
*M* *_r_* = 421.09
Monoclinic, 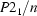

*a* = 9.829 (3) Å
*b* = 7.559 (2) Å
*c* = 15.350 (5) Å
β = 105.589 (3)°
*V* = 1098.6 (6) Å^3^
*Z* = 4
Mo *K*α radiation
μ = 8.12 mm^−1^
*T* = 296 K
0.28 × 0.26 × 0.22 mm

#### Data collection

Bruker APEXII CCD diffractometer
Absorption correction: multi-scan (*SADABS*; Bruker, 2004 ▶) *T*_min_ = 0.118, *T*_max_ = 0.168
5810 measured reflections
2028 independent reflections
1721 reflections with *I* > 2σ(*I*)
*R*_int_ = 0.038

#### Refinement

*R*[*F* ^2^ > 2σ(*F* ^2^)] = 0.031
*wR*(*F* ^2^) = 0.082
*S* = 1.08
2028 reflections
176 parameters
12 restraints
H atoms treated by a mixture of independent and constrained refinement
Δρ_max_ = 1.45 e Å^−3^
Δρ_min_ = −1.83 e Å^−3^

### 

Data collection: *APEX2* (Bruker, 2004 ▶); cell refinement: *SAINT* (Bruker, 2004 ▶); data reduction: *SAINT*; program(s) used to solve structure: *SHELXS97* (Sheldrick, 2008 ▶); program(s) used to refine structure: *SHELXL97* (Sheldrick, 2008 ▶); molecular graphics: *SHELXTL* (Sheldrick, 2008 ▶); software used to prepare material for publication: *SHELXTL*.

## Supplementary Material

Crystal structure: contains datablocks I, global. DOI: 10.1107/S1600536811007628/pv2392sup1.cif

Structure factors: contains datablocks I. DOI: 10.1107/S1600536811007628/pv2392Isup2.hkl

Additional supplementary materials:  crystallographic information; 3D view; checkCIF report

## Figures and Tables

**Table 1:** Hydrogen-bond geometry (Å, °)

*D*—H⋯*A*	*D*—H	H⋯*A*	*D*⋯*A*	*D*—H⋯*A*
O9—H8*W*⋯O2^i^	0.88	2.54	3.111 (6)	123
O9—H7*W*⋯O5^ii^	0.83	2.03	2.694 (6)	137
O8—H6*W*⋯O9^iii^	0.95	1.73	2.679 (5)	179
O8—H5*W*⋯O9^iv^	0.90	2.27	3.068 (6)	148
O7—H4*W*⋯O5^v^	0.95	1.79	2.697 (5)	158
O7—H3*W*⋯O1^i^	0.90 (3)	1.85 (3)	2.672 (5)	151 (5)
O6—H2*W*⋯O4^vi^	0.83 (4)	2.11 (5)	2.791 (5)	139 (5)
O6—H1*W*⋯O3^vii^	0.91 (4)	1.84 (4)	2.706 (5)	156 (4)

Symmetry codes: (i) 
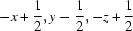
; (ii) 
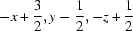
; (iii) 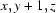; (iv) 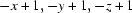; (v) 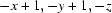; (vi) 
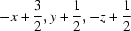
; (vii) 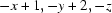.

**Fig. 1.**
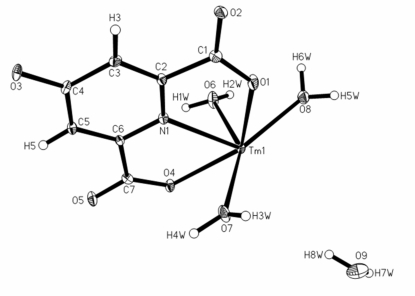
Drawing of the asymmetric unit of the title complex, showing the atom-labeling scheme. Displacement ellipsoids are drawn at the 30% probability level.

**Fig. 2.**
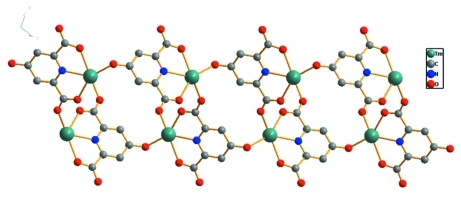
A view of the title structure along the b axis, showing the double chain.

**Fig. 3.**
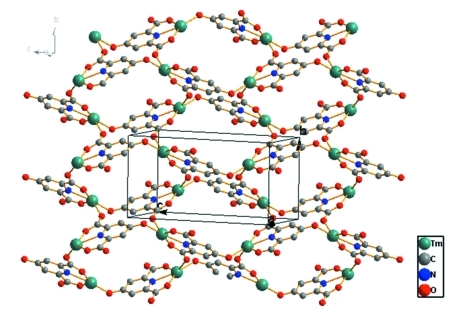
A view of the unit cell along the a axis, showing the sheet structure of the title complex; H-atoms have been excluded for clarity.

## supplementary crystallographic information

### Comment

The lanthanide coordination polymers have shown not only versatile architectures but also desirable properties, *e.g.*, luminescent, magnetic, catalytic, and gas absorption and separation properties (Qin *et al.*, 2011; Lv *et al.*, 2010). In order to extend our investigations in this field, we have designed and synthesized a novel lanthanide coordination polymer, {[Tm(C_7_H_2_NO_5_)(H_2_O)_3_].H_2_O}*_n_*, by choosing 4-oxidopyridine-2,6-dicarboxylicacid as a functional ligand, and report its crystal structure in this paper.

The title compound is isotypic with its Dy (Gao *et al.*, 2006) and Eu (Lv *et al.*, 2010) analogues. The asymmetrical unit of the the title complex contains a Tm(III) ion, a 4-oxidopyridine-2,6-dicarboxylate anion, three coordinated water molecules, and a molecule of water of crystallization (Fig. 1). The Tm atom is eight-coordinated by seven oxygen atoms from three anions and three coordinated water molecules and by a nitrogen atom from a tridentate anion (the other two anions are monodentate), forming a distorted bicapped trigonal-prismatic coordination environment. The Tm–O bond lengths [2.263 (3) - 2.385 (3) Å] are shorter than the Tm–N bond length [2.430 (4) Å], which is in agreement with the bond lengths observed in other Tm(III) complexes (Qin *et al.*, 2011; Tian *et al.*, 2009). The anion adopts a *µ*_3_-pentadentate coordination mode. The anions bridge the adjacent Tm^III^ ions to form infinite double chains (Fig. 2). Adjacent chains are further connected by the coordination of the anions and Tm(III) ions into two-dimensional sheets (Fig. 3), which are further extended into a three-dimensional supramolecular framework through O–H···O hydrogen-bonding interactions including both coordinated and uncoordinated water molecules (Table 1).

### Experimental

To a solution of thulium(III)nitrate hexahydrate (0.139 g, 0.3 mmol) in water (5 ml) was added an aqueous solution (5 ml) of the ligand (0.060 g, 0.3 mmol) and a drop of triethylamine. The reactants were sealed in a 25-ml Teflon-lined, stainless-steel Parr bomb. The bomb was heated at 433 K for 3 days. On cooling the solution, single crystals (*ca* 70% yield) were obtained which were suitable for single crystal X-ray differaction studies.

### Refinement

The coordinated water H atoms were located in a different Fourier map and were refined with distance constraints of O–H = 0.83 (3) Å. The free water H atoms were placed at calculated positions and refined with a riding model, considering the position of oxygen atoms and the quantity of H atoms. The carbon-bound H atoms were placed in geometrically idealized positions, with C–H = 0.93 Å and constrained to ride on their respective parent atoms, with *U*iso(H) = 1.2 *U*eq(C). The final difference map was esseintially faetureless with residual electron denisty within 1.0 Å of the Th atom.

### Crystal data

**Table:**

[Tm(C_7_H_2_NO_5_)(H_2_O)_3_]·H_2_O	*F*(000) = 800
*M**_r_* = 421.09	*D*_x_ = 2.546 Mg m^−^^3^
Monoclinic, *P*2_1_/*n*	Mo *K*α radiation, λ = 0.71073 Å
Hall symbol: -P 2yn	Cell parameters from 3206 reflections
*a* = 9.829 (3) Å	θ = 2.2–28.1°
*b* = 7.559 (2) Å	µ = 8.12 mm^−^^1^
*c* = 15.350 (5) Å	*T* = 296 K
β = 105.589 (3)°	Block, colorless
*V* = 1098.6 (6) Å^3^	0.28 × 0.26 × 0.22 mm
*Z* = 4	

### Data collection

**Table:**

Bruker APEXII CCD diffractometer	2028 independent reflections
Radiation source: fine-focus sealed tube	1721 reflections with *I* > 2σ(*I*)
graphite	*R*_int_ = 0.038
φ and ω scans	θ_max_ = 25.5°, θ_min_ = 2.2°
Absorption correction: multi-scan (*SADABS*; Bruker, 2004)	*h* = −11→11
*T*_min_ = 0.118, *T*_max_ = 0.168	*k* = −9→9
5810 measured reflections	*l* = −18→11

### Refinement

**Table:**

Refinement on *F*^2^	Secondary atom site location: difference Fourier map
Least-squares matrix: full	Hydrogen site location: inferred from neighbouring sites
*R*[*F*^2^ > 2σ(*F*^2^)] = 0.031	H atoms treated by a mixture of independent and constrained refinement
*wR*(*F*^2^) = 0.082	*w* = 1/[σ^2^(*F*_o_^2^) + (0.039*P*)^2^ + 1.1506*P*] where *P* = (*F*_o_^2^ + 2*F*_c_^2^)/3
*S* = 1.08	(Δ/σ)_max_ = 0.002
2028 reflections	Δρ_max_ = 1.45 e Å^−^^3^
176 parameters	Δρ_min_ = −1.83 e Å^−^^3^
12 restraints	Extinction correction: *SHELXL97* (Sheldrick, 2008), Fc^*^=kFc[1+0.001xFc^2^λ^3^/sin(2θ)]^-1/4^
Primary atom site location: structure-invariant direct methods	Extinction coefficient: 0.0158 (6)

### Special details

**Table:**

Experimental. Anal. Calcd for C_7_H_10_TbNO_9_: C, 19.97; H, 2.39; N, 3.33. Found: C, 20.31; H, 2.47; N, 3.12.
Geometry. All e.s.d.'s (except the e.s.d. in the dihedral angle between two l.s. planes) are estimated using the full covariance matrix. The cell e.s.d.'s are taken into account individually in the estimation of e.s.d.'s in distances, angles and torsion angles; correlations between e.s.d.'s in cell parameters are only used when they are defined by crystal symmetry. An approximate (isotropic) treatment of cell e.s.d.'s is used for estimating e.s.d.'s involving l.s. planes.
Refinement. Refinement of *F*^2^ against ALL reflections. The weighted *R*-factor *wR* and goodness of fit *S* are based on *F*^2^, conventional *R*-factors *R* are based on *F*, with *F* set to zero for negative *F*^2^. The threshold expression of *F*^2^ > σ(*F*^2^) is used only for calculating *R*-factors(gt) *etc*. and is not relevant to the choice of reflections for refinement. *R*-factors based on *F*^2^ are statistically about twice as large as those based on *F*, and *R*- factors based on ALL data will be even larger.

### Fractional atomic coordinates and isotropic or equivalent isotropic displacement parameters (Å^2^)

**Table:**

	*x*	*y*	*z*	*U*_iso_*/*U*_eq_	
Tm1	0.498130 (19)	0.67912 (3)	0.252028 (12)	0.00947 (16)	
C1	0.2175 (5)	0.9137 (7)	0.1631 (3)	0.0156 (11)	
C2	0.2755 (5)	0.8881 (6)	0.0829 (3)	0.0127 (10)	
C3	0.2046 (5)	0.9447 (6)	−0.0015 (3)	0.0155 (11)	
H3	0.1204	1.0072	−0.0105	0.019*	
C4	0.2614 (5)	0.9069 (6)	−0.0753 (3)	0.0121 (10)	
C5	0.3907 (5)	0.8160 (6)	−0.0544 (3)	0.0132 (11)	
H5	0.4336	0.7888	−0.0999	0.016*	
C6	0.4537 (5)	0.7675 (6)	0.0335 (3)	0.0123 (10)	
C7	0.5906 (5)	0.6682 (6)	0.0630 (4)	0.0132 (11)	
N1	0.3961 (5)	0.8002 (5)	0.1020 (3)	0.0125 (9)	
O1	0.2806 (4)	0.8294 (5)	0.2333 (3)	0.0242 (10)	
O2	0.1121 (4)	1.0091 (5)	0.1562 (2)	0.0190 (8)	
O3	0.1933 (4)	0.9514 (5)	−0.1587 (2)	0.0173 (8)	
O4	0.6311 (4)	0.6267 (5)	0.1454 (2)	0.0168 (8)	
O5	0.6587 (4)	0.6359 (5)	0.0068 (2)	0.0212 (9)	
O6	0.6355 (4)	0.9322 (5)	0.2582 (3)	0.0251 (9)	
O7	0.3975 (5)	0.4312 (5)	0.1720 (3)	0.0269 (10)	
H4W	0.3923	0.3823	0.1140	0.032*	
O8	0.5064 (5)	0.8348 (4)	0.3875 (3)	0.0232 (9)	
H5W	0.4593	0.8051	0.4279	0.028*	
H6W	0.5247	0.9565	0.4025	0.028*	
O9	0.5599 (5)	0.1768 (5)	0.4284 (3)	0.0386 (12)	
H7W	0.6306	0.1698	0.4728	0.046*	
H8W	0.5610	0.2542	0.3853	0.046*	
H1W	0.689 (5)	0.940 (7)	0.218 (3)	0.018 (14)*	
H2W	0.689 (5)	0.964 (7)	0.307 (3)	0.017 (16)*	
H3W	0.318 (5)	0.393 (6)	0.184 (3)	0.012 (13)*	

### Atomic displacement parameters (Å^2^)

**Table:**

	*U*^11^	*U*^22^	*U*^33^	*U*^12^	*U*^13^	*U*^23^
Tm1	0.0065 (2)	0.0139 (2)	0.0060 (2)	0.00015 (7)	−0.00175 (13)	0.00069 (7)
C1	0.010 (3)	0.020 (3)	0.014 (3)	0.002 (2)	−0.002 (2)	−0.001 (2)
C2	0.010 (3)	0.018 (2)	0.010 (3)	0.001 (2)	0.001 (2)	−0.001 (2)
C3	0.011 (3)	0.018 (3)	0.016 (3)	0.004 (2)	0.001 (2)	0.003 (2)
C4	0.008 (3)	0.016 (2)	0.011 (3)	−0.0047 (19)	−0.001 (2)	0.003 (2)
C5	0.010 (3)	0.019 (3)	0.010 (3)	−0.0025 (19)	0.001 (2)	−0.0009 (19)
C6	0.014 (3)	0.013 (2)	0.010 (3)	0.000 (2)	0.003 (2)	−0.001 (2)
C7	0.007 (3)	0.015 (2)	0.016 (3)	−0.0042 (19)	0.001 (2)	−0.004 (2)
N1	0.007 (2)	0.019 (2)	0.011 (2)	0.0006 (17)	0.0003 (19)	0.0006 (16)
O1	0.019 (2)	0.041 (2)	0.012 (2)	0.0165 (16)	0.0038 (18)	0.0091 (16)
O2	0.017 (2)	0.0239 (19)	0.015 (2)	0.0084 (16)	0.0027 (16)	0.0000 (15)
O3	0.015 (2)	0.025 (2)	0.008 (2)	−0.0046 (15)	−0.0036 (16)	0.0028 (14)
O4	0.013 (2)	0.0250 (19)	0.011 (2)	0.0046 (16)	0.0015 (15)	0.0032 (15)
O5	0.017 (2)	0.035 (2)	0.011 (2)	0.0074 (17)	0.0034 (16)	0.0002 (16)
O6	0.028 (3)	0.032 (2)	0.015 (2)	−0.0193 (18)	0.006 (2)	−0.0063 (18)
O7	0.037 (3)	0.030 (2)	0.018 (2)	−0.0158 (19)	0.013 (2)	−0.0105 (17)
O8	0.031 (3)	0.022 (2)	0.018 (2)	−0.0030 (16)	0.008 (2)	−0.0017 (15)
O9	0.027 (3)	0.027 (2)	0.055 (3)	−0.0055 (17)	−0.001 (2)	−0.0030 (19)

### Geometric parameters (Å, °)

**Table:**

Tm1—O3^i^	2.263 (3)	C5—C6	1.374 (7)
Tm1—O7	2.312 (4)	C5—H5	0.9300
Tm1—O6	2.329 (4)	C6—N1	1.345 (6)
Tm1—O1	2.369 (4)	C6—C7	1.501 (7)
Tm1—O8	2.372 (4)	C7—O5	1.250 (6)
Tm1—O2^ii^	2.372 (3)	C7—O4	1.260 (6)
Tm1—O4	2.385 (3)	O2—Tm1^iii^	2.372 (3)
Tm1—N1	2.430 (4)	O3—Tm1^iv^	2.263 (3)
C1—O2	1.243 (6)	O6—H1W	0.91 (4)
C1—O1	1.262 (6)	O6—H2W	0.83 (4)
C1—C2	1.502 (7)	O7—H4W	0.9517
C2—N1	1.321 (6)	O7—H3W	0.90 (3)
C2—C3	1.364 (7)	O8—H5W	0.8963
C3—C4	1.421 (7)	O8—H6W	0.9533
C3—H3	0.9300	O9—H7W	0.8345
C4—O3	1.318 (6)	O9—H8W	0.8850
C4—C5	1.404 (7)		
			
O3^i^—Tm1—O7	98.07 (13)	C3—C2—C1	121.9 (5)
O3^i^—Tm1—O6	86.88 (14)	C2—C3—C4	119.0 (5)
O7—Tm1—O6	147.98 (14)	C2—C3—H3	120.5
O3^i^—Tm1—O1	150.82 (12)	C4—C3—H3	120.5
O7—Tm1—O1	94.66 (15)	O3—C4—C5	122.6 (4)
O6—Tm1—O1	96.05 (14)	O3—C4—C3	121.2 (5)
O3^i^—Tm1—O8	82.05 (13)	C5—C4—C3	116.2 (4)
O7—Tm1—O8	141.00 (13)	C6—C5—C4	119.8 (5)
O6—Tm1—O8	70.96 (13)	C6—C5—H5	120.1
O1—Tm1—O8	71.66 (14)	C4—C5—H5	120.1
O3^i^—Tm1—O2^ii^	81.52 (13)	N1—C6—C5	123.1 (5)
O7—Tm1—O2^ii^	71.23 (12)	N1—C6—C7	112.8 (4)
O6—Tm1—O2^ii^	140.63 (13)	C5—C6—C7	124.1 (4)
O1—Tm1—O2^ii^	77.90 (13)	O5—C7—O4	124.2 (5)
O8—Tm1—O2^ii^	70.24 (12)	O5—C7—C6	119.5 (5)
O3^i^—Tm1—O4	79.12 (12)	O4—C7—C6	116.3 (4)
O7—Tm1—O4	74.67 (13)	C2—N1—C6	117.4 (4)
O6—Tm1—O4	75.29 (13)	C2—N1—Tm1	121.1 (3)
O1—Tm1—O4	129.75 (12)	C6—N1—Tm1	121.3 (3)
O8—Tm1—O4	141.98 (13)	C1—O1—Tm1	124.5 (3)
O2^ii^—Tm1—O4	137.63 (11)	C1—O2—Tm1^iii^	140.1 (3)
O3^i^—Tm1—N1	143.61 (13)	C4—O3—Tm1^iv^	127.1 (3)
O7—Tm1—N1	78.04 (14)	C7—O4—Tm1	124.4 (3)
O6—Tm1—N1	79.41 (14)	Tm1—O6—H1W	117 (3)
O1—Tm1—N1	64.89 (13)	Tm1—O6—H2W	120 (4)
O8—Tm1—N1	123.52 (13)	H1W—O6—H2W	104 (4)
O2^ii^—Tm1—N1	128.94 (14)	Tm1—O7—H4W	136.0
O4—Tm1—N1	64.86 (13)	Tm1—O7—H3W	115 (3)
O2—C1—O1	124.9 (5)	H4W—O7—H3W	103.9
O2—C1—C2	119.8 (4)	Tm1—O8—H5W	125.4
O1—C1—C2	115.3 (4)	Tm1—O8—H6W	130.5
N1—C2—C3	124.6 (5)	H5W—O8—H6W	100.0
N1—C2—C1	113.5 (4)	H7W—O9—H8W	118.5
			
O2—C1—C2—N1	172.9 (5)	O4—Tm1—N1—C2	180.0 (4)
O1—C1—C2—N1	−8.8 (6)	O3^i^—Tm1—N1—C6	14.6 (5)
O2—C1—C2—C3	−9.9 (7)	O7—Tm1—N1—C6	−72.8 (4)
O1—C1—C2—C3	168.4 (5)	O6—Tm1—N1—C6	84.3 (4)
N1—C2—C3—C4	0.6 (7)	O1—Tm1—N1—C6	−173.8 (4)
C1—C2—C3—C4	−176.2 (4)	O8—Tm1—N1—C6	142.8 (3)
C2—C3—C4—O3	176.7 (4)	O2^ii^—Tm1—N1—C6	−126.4 (4)
C2—C3—C4—C5	−1.6 (7)	O4—Tm1—N1—C6	5.7 (3)
O3—C4—C5—C6	−177.6 (4)	O2—C1—O1—Tm1	−171.5 (4)
C3—C4—C5—C6	0.7 (7)	C2—C1—O1—Tm1	10.3 (6)
C4—C5—C6—N1	1.2 (7)	O3^i^—Tm1—O1—C1	163.5 (4)
C4—C5—C6—C7	179.2 (4)	O7—Tm1—O1—C1	−80.7 (4)
N1—C6—C7—O5	−176.9 (4)	O6—Tm1—O1—C1	69.1 (4)
C5—C6—C7—O5	5.0 (7)	O8—Tm1—O1—C1	136.7 (4)
N1—C6—C7—O4	1.3 (6)	O2^ii^—Tm1—O1—C1	−150.3 (4)
C5—C6—C7—O4	−176.9 (4)	O4—Tm1—O1—C1	−6.8 (5)
C3—C2—N1—C6	1.3 (7)	N1—Tm1—O1—C1	−6.2 (4)
C1—C2—N1—C6	178.4 (4)	O1—C1—O2—Tm1^iii^	25.6 (9)
C3—C2—N1—Tm1	−173.2 (4)	C2—C1—O2—Tm1^iii^	−156.3 (4)
C1—C2—N1—Tm1	3.9 (6)	C5—C4—O3—Tm1^iv^	104.5 (5)
C5—C6—N1—C2	−2.2 (7)	C3—C4—O3—Tm1^iv^	−73.7 (5)
C7—C6—N1—C2	179.6 (4)	O5—C7—O4—Tm1	−177.8 (3)
C5—C6—N1—Tm1	172.3 (4)	C6—C7—O4—Tm1	4.1 (6)
C7—C6—N1—Tm1	−5.9 (5)	O3^i^—Tm1—O4—C7	−179.8 (4)
O3^i^—Tm1—N1—C2	−171.1 (3)	O7—Tm1—O4—C7	78.6 (4)
O7—Tm1—N1—C2	101.5 (4)	O6—Tm1—O4—C7	−90.2 (4)
O6—Tm1—N1—C2	−101.4 (4)	O1—Tm1—O4—C7	−4.6 (4)
O1—Tm1—N1—C2	0.5 (4)	O8—Tm1—O4—C7	−118.1 (4)
O8—Tm1—N1—C2	−42.9 (4)	O2^ii^—Tm1—O4—C7	115.9 (4)
O2^ii^—Tm1—N1—C2	47.9 (4)	N1—Tm1—O4—C7	−5.1 (3)

Symmetry codes: (i) *x*+1/2, −*y*+3/2, *z*+1/2; (ii) −*x*+1/2, *y*−1/2, −*z*+1/2; (iii) −*x*+1/2, *y*+1/2, −*z*+1/2; (iv) *x*−1/2, −*y*+3/2, *z*−1/2.

### Hydrogen-bond geometry (Å, °)

**Table:**

*D*—H···*A*	*D*—H	H···*A*	*D*···*A*	*D*—H···*A*
O9—H8W···O2^ii^	0.88	2.54	3.111 (6)	123
O9—H7W···O5^v^	0.83	2.03	2.694 (6)	137
O8—H6W···O9^vi^	0.95	1.73	2.679 (5)	179
O8—H5W···O9^vii^	0.90	2.27	3.068 (6)	148
O7—H4W···O5^viii^	0.95	1.79	2.697 (5)	158
O7—H3W···O1^ii^	0.90 (3)	1.85 (3)	2.672 (5)	151 (5)
O6—H2W···O4^ix^	0.83 (4)	2.11 (5)	2.791 (5)	139 (5)
O6—H1W···O3^x^	0.91 (4)	1.84 (4)	2.706 (5)	156 (4)

Symmetry codes: (ii) −*x*+1/2, *y*−1/2, −*z*+1/2; (v) −*x*+3/2, *y*−1/2, −*z*+1/2; (vi) *x*, *y*+1, *z*; (vii) −*x*+1, −*y*+1, −*z*+1; (viii) −*x*+1, −*y*+1, −*z*; (ix) −*x*+3/2, *y*+1/2, −*z*+1/2; (x) −*x*+1, −*y*+2, −*z*.
